# Intercultural Usage of Mori Folium: Comparison Review from a Korean Medical Perspective

**DOI:** 10.1155/2015/379268

**Published:** 2015-10-11

**Authors:** Byungjin Joh, Eun Sang Jeon, Su Hye Lim, Yu Lee Park, Wansu Park, Han Chae

**Affiliations:** ^1^Korean Medicine Global Center, Association of Korean Medicine, Seoul 07525, Republic of Korea; ^2^Department of Pharmacognosy, Faculty of Pharmacy, Istanbul University, 34452 Istanbul, Turkey; ^3^Department of Preventive Medicine, College of Korean Medicine, Kyung Hee University, Seoul 02447, Republic of Korea; ^4^Department of Pathology, College of Korean Medicine, Gachon University, Seongnam 13120, Republic of Korea; ^5^Division of Longevity and Biofunctional Medicine, School of Korean Medicine, Pusan National University, Busan 50612, Republic of Korea

## Abstract

*Objectives*. A review on studies related to the use of Mori folium, the leaves of *Morus alba*, was conducted with the goal of identifying new clinical applications in Korean medicine. *Methods*. Global literature search was conducted using three electronic databases up to January 2015 with the term *Morus alba* and its Korean terms. KM literatures including textbooks and standard pharmacopoeia were separately hand-searched and reviewed to provide comparison. Data were extracted according to predetermined criteria, and clinical uses were standardized with ICD-10 categories. *Results*. 159 potentially relevant studies were identified, and 18 articles including 12 ethnopharmacologic and 6 clinical studies were finally included in this analysis. Ethnopharmacologic studies from 8 countries provided 17 clinical uses. We found that five out of six clinical trials were related to diabetes and suggested a moderate short-term to mild long-term effect. And 43 Korean texts also provided 156 clinical uses in 35 categories including ocular and respiratory disorders. *Discussion and Conclusions*. Though majority of the clinical uses were also found in Korean medicine literature, treatment of infertility, jaundice, cognitive disorder, and hyperpigmentation was found to be effective and diabetes with *Morus alba* was recognized to have clinical importance.

## 1. Introduction

*Morus alba *Linne is a deciduous tree that belongs to the Moraceae family. Mulberry trees are usually grown throughout the world including Korea and have been used in many ways in traditional medicine for a long time [1].

Mori folium is also used agriculturally for feeding the silkworms, and in many countries like Turkey and Greece, Mori fructus has a role in food supply [2]. In Asia, Africa, Europe, and South and North America,* Morus alba* is widely used to treat diseases for its antimicrobial and antioxidant properties [3, 4], however, mainly for antidiabetic, immunomodulatory, antimicrobial, antioxidant, and anticancer purposes [5]. The major phytochemical component of Mori folium is rutin, and Mulberry leaves are known to have antipyretic, antitussive, anti-inflammatory, and hepatoprotective effects [6]. Mori folium is also frequently used in Korea for treating common cold, cough, headaches, and red swollen eyes [6]. Korea has a dual medical system of conventional Western medicine and indigenously developed traditional Korean medicine (KM) and tries to combine these for more integrative medical services [7]. Traditional Korean medicine has a clinical experience of more than five thousand years and has influence of traditional Chinese medicine with more emphasis on Person-Centered Medicine [8]. A 2014 nationwide survey by the Korean Ministry of Health and Welfare found that 27.1% of the general respondents received KM treatment within the past 3 years, and 68.8% were found to have intention to use KM for treatment [9]. Considering that Mori folium is an abundant, economical, and versatile herbal resource, expanding its clinical application range in the KM system would therefore be beneficial both economically and medically. There is a difference in applications of Mori folium in KM and other medical systems, but studies addressing this gap are not available yet.

The current study thus aims to identify potential clinical areas of additional use of Mori folium in KM practice by reviewing ethnopharmacologic and clinical literature from other medical systems and comparing the results with a narrative review of Korean medical literatures. The specific procedures and methods are described in the following section.

## 2. Materials and Methods

### 2.1. Search Strategy and Data Sources

Three electronic databases including PubMed (http://www.ncbi.nlm.nih.gov/pubmed/), ScienceDirect (http://www.sciencedirect.com/), and Korean studies Information Service System (KISS, http://kiss.kstudy.com/) were searched for materials made available up to January 2015. The keywords entered were “*Morus alba*” in all databases and the Korean terms of Mori folium and* Morus alba* in KISS.

Herbs are usually administered as a multiherbal prescription and seldom used as single herb in KM. Assessment of full texts provided only one Korean ethnopharmacologic study of Mori folium in single herb. Since this was deemed insufficient to serve as a basis for comparison, a narrative review on effect of Mori folium in KM was separately performed.

### 2.2. Article Selection and Data Extraction

#### 2.2.1. Inclusion and Exclusion Criteria

Ethnopharmacologic studies providing uses and effects attributed specifically to Mori folium alone were included, but multiherbal prescriptions were excluded. Studies providing secondhand information only were excluded to avoid duplication.

Clinical studies on isolated substances from Mori folium were excluded for this review since they cannot represent the whole range of substances present in the material as used in KM practice. Research on Mori folium products with certain fortified substances was included only when new substances were not included. Case reports were excluded since they were deemed not to represent usage. Other disputes were settled through consensus among authors.

Out of 3,421 articles identified in the search process, one KM ethnopharmacologic field study was analyzed separately in the KM narrative review, and 18 articles were included in this review.

#### 2.2.2. Data Extraction

All articles were independently reviewed by three reviewers (Jeon, Lim, and Joh) and data was extracted by predefined criteria (see Table 1). These criteria were modified from previous reviews on ethnopharmacology and/or clinical trials of similar nature [10, 11]. Due to disparities in description of quality and cultural context, uses were standardized and coded as the closest matching category listed on the 10th International Classification of Diseases (ICD-10) [12]. Only firsthand data was used from each study.

#### 2.2.3. Methodological Quality Assessment

There is currently no validated tool for assessing ethnopharmacologic research quality and methodological quality of clinical studies was assessed with JADAD scale [13]. The JADAD scale has been used for assessing the methodological quality of randomized controlled clinical trials depending on its description of randomization, blinding, and others with score range of 0 to 5 [13]. Studies with 3–5 points were regarded as good methodological quality and 0–2 points were poor methodological quality.

#### 2.2.4. Data Analysis

The data analysis was conducted using the following process. Clinical uses and nationality or cultural origin of research subjects in ethnopharmacologic studies were analyzed and summarized. Clinical trials were first grouped according to ICD-10 subchapters of usage. Subsequently, exploration of the significant factors for the clinical characteristics of each group was conducted.

### 2.3. KM Literature Review

A literature review was separately performed as previously mentioned in Section 2.1. KM pharmacology textbooks currently used in one or more colleges and 10 classics recognized by Korean Food and Drug Administration as standard pharmacopoeias were searched for uses of Mori folium. Literature from the previous database search containing first- or secondhand information on KM use of Mori folium, including one ethnopharmacologic field study, was also retrieved for review in this part. Furthermore, the references in all the located articles were manually searched for additional relevant article search. Data extraction was performed according to the predefined criteria in Table 1, with the exception of originating culture since all pertained to KM.

Clinical usage data attributed to Mori folium were extracted, but effects of multiherbal prescriptions were excluded. To preserve the cultural intonations of the data, uses were first standardized into 2nd Korean Standard Classification of Diseases-Oriental Medicine (KCD-OM2) codes which employ KM diagnostic terminology and then translated into pertaining ICD-10 category codes based on the diagnosis matching chart of KCD-OM2. Closest matches were used in both coding steps. The same ICD-10 categories appearing multiple times in a single text were counted as one use.

## 3. Results

**Figure 1:**
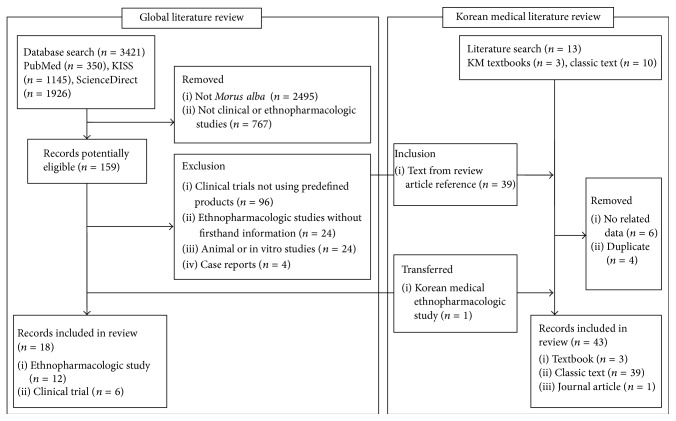
Data extraction process of the current review.

3421 potentially relevant articles for the global literature review were found on initial search of databases.  Figure 1 shows the data extraction process, and the results are summarized in Tables 2 and 3. The detailed analyses were presented in the following sections.

### 3.1. Ethnopharmacologic Research

17 records of clinical uses in 13 categories were found from 12 non-Korean ethnopharmacologic studies with 8 cultural origins.  Table 2 provides a summary for these.

Mori folium was used in multiple studies for respiratory tract disorders at Pakistan. It was used in 3 instances such as an expectorant and sore throat treatment [14] and to relieve cough due to throat pain [15]. Two studies described the clinical use for ocular disorders including relieving effect on sore and inflamed eyes [14] and blindness treatment [16]. Substances in Mori folium such as rutin, choline, and folic acid have anti-inflammatory effects which might be a reason for these clinical effects [6].

Other uses include dizziness and vertigo treatment, antipyretic, analgesic [14], antivenom [17], antihypertensive [18], anti-infertility [19], antidiabetic [20], anticancer [21], reducer [22], neuritis treatment [23], jaundice treatment [24], and hyperpigmentation treatment [25]. Chlorogenic acid has neuroprotective functions which might be in play [26]. Eugenol is a local antiseptic and anaesthetic [27] and has been demonstrated to have anticancer activities against certain human cancer cell lines in vitro and in vivo [28]. Chlorogenic acid plays a role in cancer prevention and protection in animal models [29], possibly through increasing DNA repair rates. Folic acid is an important substance in the reproductive process of both men and women [30]. Folic acid also plays a controversial role in cancer patients; diets high in folate are associated with decreased risk of colorectal cancer [31], but the quickly multiplying cancer cells require folate for growth and reducing its availability to cancer cells is a major pathway in mechanisms of commonly used chemotherapy agents such as methotrexate. The actions of these substances might provide at least some parts of medicinal effects of Mori folium in humans.

### 3.2. Clinical Trials

Six clinical trials within predetermined criteria were found and had small or moderate sample size. Five studies were on diabetes or postprandial glucose suppression, and one study was on cognitive function.  Table 3 provides a summary of these clinical trials. Various Mori folium-derived products were used around the globe in herbal supplements for blood glucose management, and the present results reflected this fact. The key active substance for this application is the glucose analogue 1-deoxynojirimycin (DNJ). It is the most abundant iminosugar in Mori folium and is an *α*-glycosidase inhibitor [32]. HPLC fluorescent detection of DNJ in Mori folium from* Morus alba* L. showed the content to be at 0.12% [33]. Most of the studies attempted to use materials with higher concentrations of DNJ, ranging from 0.36% to 1.5% of dry weight. Single dose studies for postprandial glucose suppression had more robust results, while long-term studies on plasma glucose provided weaker results.

The single study on cognitive function was a randomized clinical trial with poor methodological quality. It suggested a possible improvement of cognition in mild cognitive impairment patients [34], but the small number of subjects and poor methodological quality made the evidence weak.

### 3.3. Korean Medical Literature Review

One ethnopharmacologic study provided a record of treatment of sore throat [35]. 40 classics and contemporary texts retrieved from the references of a literature review on Mori folium provided the majority of clinical uses [36]. 156 mentions of clinical uses in 35 ICD-10 categories were found from 43 texts. The most prevalent uses were for ocular disorders (*n* = 33), respiratory disorders (*n* = 24), analgesic purposes (*n* = 16), treating excessive sweating (*n* = 15), joint disorders (*n* = 11), and gastrointestinal tract disorders (*n* = 11).  Table 4 provides a summary of the literature review.

## 4. Discussion

The current study examined global and Korean literature on medical use of Mori folium to discover additional clinical applications in traditional Korean medicine. In traditional Korean medicine (Table 4), Mori folium clears heat of the lungs which can be understood as the inflammation or congestion in the upper body, respiratory system, and skin. The anti-inflammatory effects might explain the clinical reports in the world, which showed antipyretic, analgesic, antivenom, antihypertensive, and anti-infertility properties that can be used in respiratory and ocular disorders, neuritis, jaundice, and hyperpigmentation (Tables 2 and 3). These clinical uses can be easily understood when we compare the reports using the standardized ICD-10 codes.

However there are some other clinical uses uniquely for traditional Korean medicine including treatment of hyperhidrosis, gastrointestinal tract, and joint disorders and use as hair tonic that can be adopted in other medical systems. Along with these, treatments of infertility [19], jaundice [24], cognitive disorder [34], and hyperpigmentation [25] were not or seldom described in Korean medical classics and texts (Tables 3 and 4).

There have been many clinical reports of Mori folium on blood glucose and diabetes (Tables 3 and 4). Though different research settings and low research quality of these reports lower the reliability of these, they showed strong possibility of dose-dependent suppression of postprandial glucose elevation that can have long-term effect on patients with severe glucose metabolism impairment. There still lies a need for well-designed clinical study, which may provide pivotal methods for diabetes treatment.

As for the summary and analysis in this study, the authors coded reported clinical uses into ICD-10 categories to compare their clinical effects. Though ethnopharmacologic studies sometimes have used ICD-10 chapters to categorize medicinal effects [37–39], the present study used categories since chapters and subchapters were too broad to incorporate all the clinical uses of Mori folium in the world. Also, the classification into chapters would cause overrepresentation of the system chapter since many ethnopharmacologic records were just simple mention of symptom names.

Although the ICD-10 coding system has universal use and usefulness for comparing studies from the world in scientific ways, there might be inevitable losses during the translation due to the nature of Western medicine underneath the coding system. For example, ICD-10 does not differentiate fever caused by liver heat and kidney deficiency, which has different accompanying signs and symptoms, and warrants completely different ways of treatment. Therefore, it should be noted that the findings in this study should be thoroughly explored with respect to their originating culture or nationality before the clinical application. And, the indicators of clinical importance and reliability were also not provided for the majority of studies. Therefore, all the potential clinical uses found in this review should be reexamined with further studies regardless of citation frequencies in here.

In this study, we excluded the clinical reports with multiherbal decoction including Mori folium as one of the ingredients and rather included description from medical textbooks, classics, and pharmacopoeia, since clinical action and effectiveness of a given herb may vary according to its role in the prescription and so it is hard to analyze its clinical usefulness. As the medical herbs are usually used as a multiherbal decoction in traditional Korean and Chinese medicine, there would be a possibility that Korean ethnopharmacologic usage and Chinese clinical studies might be unsatisfactory in this review [40]. Also, limitation with accessible clinical research databases might also have happened as for the local literatures with other languages not included here.

In conclusion, we performed a review on clinical use of Mori folium with three database and traditional Korean medical textbooks and pharmacopoeia and analyzed its clinical use with ICD-10 code. From 159 relevant studies and 17 clinical usages, infertility, jaundice, cognitive disorder, and hyperpigmentation were identified as potential clinical uses, and diabetes was the one deserving more emphasis. This study would contribute to the thorough understanding on the clinical usefulness of* Morus alba* and Mori folium with carefully designed researches on its clinical applications.

## Figures and Tables

**Table 1:** Predefined criteria for data extraction.

Ethnopharmacologic studies				
Study & text details	Uses^†^	Culture or nationality		
ID (e.g., first author) Year of publication	Disorder treatment Symptom treatment Preventive purposes Tonic purposes	Nationality of subjects		

Clinical studies				
Study details	Uses	Population	Material	Outcome measures & results

ID (e.g., first author) Year of publication Study design Research quality	Disorder treatment Symptom treatment Preventive purposes Tonic purposes	Diagnosis Clinical setting Sample sizes (cases and controls or cohort size, including recruitment success rates) Other important participant characteristics	Material used (intervention & control) Dosage Administration schedule	Primary outcome measures Laboratory results Questionnaire scores Physical symptoms Incidence rates of given event within population

^†^Only single use of Mori folium herb was retrieved.

**Table 2:** Ethnopharmacologic studies on Mori folium.

Author (year)	Relevant ICD-10 category	Description of use in original article	Culture/nationality
Menale and Muoio (2014) [18]	I10 essential (primary) hypertension	High blood pressure	Italy

Bibi et al. (2014) [15]	R05 cough	The leaves were boiled in water (Joshanda) and given for cough due to throat pain	Pakistan

Ullah et al. (2013) [14]	J40 bronchitis, not specified as acute or chronic R50 fever H57 other disorders of eye and adnexa J03 acute tonsillitis R51 headache R42 dizziness and giddiness	White mulberry leaves are expectorant, encouraging the loosing and coughing up of catarrh, and are prescribed in China as a treatment for coughs. The leaves are also taken to treat fever, sore and inflamed eyes, sore throats, headache, dizziness, and vertigo	Pakistan

Tetik et al. (2013) [22]	n/a	Reducing^†^	Turkey

Gakuya et al. (2013) [16]	H54 visual impairment including blindness	Blindness	Kenya

Oliveira et al. (2012) [21]	C80 malignant neoplasm, without specification of site	Cancer	Brazil

Sathiyaraj et al. (2012) [19]	N46 male infertility^††^	Anti-infertility	India

Sharma et al. (2012) [24]	R17 unspecified jaundice	Leaf juice mixed with one cup of curd is given once a day till the patient is cured	India

Adhikari et al. (2008) [25]	L81 other disorders of pigmentation	Hyperpigmentation	Nepal

Au et al. (2008) [23]	M79 other soft tissue disorders	Neuritis	China

Arýkan et al. (2009) [20]	E10 type 1 diabetes mellitus	Type 1 diabetes in children	Turkey

Samy et al. (2008) [17]	T63 toxic effect of contact with venomous animals	Snakebite treatment	India

^†^It might refer to weight reduction, fetal reduction, and use in conjunction with orthopedic reduction, fever reduction, or cicatrizer.

^††^Gender is not explicitly mentioned, but study describes male infertility.

**Table 3:** Clinical trials using Mori folium or derived products.

Author & year	Study type	Condition & ICD-10	Participants & sample size	Material used	Dosage & duration	JADAD score	Primary outcome measures	Main results
Banu et al. (2015) [41]	CC	Type 2 diabetes (E11)	48 type 2 diabetics on oral medication (28 intervention + 20 black tea placebo)	Tea (*Mulbericha green* variety)	1 cup (70 mL), single dose	—	Plasma glucose change before meal with tea and 90 minutes afterwards	Plasma glucose levels in control and intervention groups before meal and 90 minutes after being changed from 178.55 ± 35.61 to 287.20 ± 56.37 (control) and 153.50 ± 48.10 to 210.21 ± 58.73 (intervention) (*p* < 0.001, effect size 1.31)

Chung et al. (2013) [42]	RCT	Glucose suppression (n/a)	50 healthy people between ages 20 and 50 (10 + 10 + 10 + 10 + 10)^†^	Aqueous extract (0.36% DNJ)	0 g, 1.25 g, 2.5 g, and 5 g extract, single dose	3 (1 + 0 + 1 + 0 + 1)	Plasma glucose level at 0, 15, 30, 60, 90, and 120 minutes after maltose intake	Intake of 2.5 g or 5 g with maltose suppressed glucose elevation significantly compared to control (*p* < 0.05). 5 g before and after treatment showed no significant difference.

Asai et al. (2011) [32]	RCT + CR^††^	Diabetes NOS (E14)	76 adults with 110–140 mg/dL FPG (38 intervention + 38 placebo)	Enriched extract (1.5% DNJ)	6 mg DNJ equivalent extract, t.i.d., 12 weeks	4 (1 + 0 + 1 + 1 + 1)	Fasting plasma glucose, insulin, HbA1c, glycated albumin, 1,5-anhydroglucitol at weeks 0, 4, 8, 12, and 16	No significant difference was found between groups except for 1, 5 AG at weeks 8 & 12 (*p* < 0.05), but difference was not maintained after treatment at week 16.

Srichaikul (2012) [34]	RCT	Other mental disorders (F06)	20 women with mild cognitive impairment (5 + 5 + 5 + 5)^†††^	Extract (*Burirum-60* variety)	200 mg, q.d., 3 months	2 (1 + 0 + 1 + 0 + 0)	SAGE, MMSE scale score changes at start and end of treatment period	Mean SAGE score rank changed from 14.1, 12.7, 7.3, and 7.9 to 17.70, 8.7, 11.0, and 4.6. Mean MMSE score rank changed from 17.4, 10.5, 6.7, and 7.4 to 17.6, 10.1, 8.5, and 5.8.

Nakamura et al. (2009) [43]	CR	Glucose suppression (n/a)	10 healthy volunteers	Ethanol extract (0.77% DNJ)	1.2 or 3 g, single dose	—	Plasma glucose (Glu.) & insulin (Ins.) (every 30 minutes for 3 hours)	Glucose and insulin elevation was suppressed in 1.2 and 3 g group compared to control at different time points (1.2 g Glu.: 30, 120 min/Ins.: 30 min/3 g Glu.: 30.90, 120 min/Ins.: 30 min/all *p* < 0.05)

Yang and Han (2006) [44]	RCT	Type 2 diabetes (E11)	25 type 2 diabetics on oral medication (14 intervention + 9 control)	Aqueous extract (0.5% DNJ)	500 mg b.i.d., 12 weeks	4 (1 + 0 + 1 + 1 + 1)	Fasting plasma glucose, HbA1c, serum lipids at start and end of treatment period	HbA1c, LDL-C, and TG decreased in intervention compared to control (*p* < 0.05, 0.05, 0.01). FBS >140 mg/dL or HbA1c >8% subjects showed FBS or HbA1c decrease (<0.05, <0.05) but <140 mg/dL or <8% subjects did not.

RCT, randomized controlled trial; CR, crossover; CC, case-controlled. All medications were taken orally.

^†^Group 1: 0 g extract with 75 g maltose. Group 2: 1.25 g extract with 75 g maltose. Group 3: 2.5 g extract with 75 g maltose. Group 4: 5 g extract with 75 g maltose. Group 5: 5 g extract 30 minutes before 75 g maltose. Group 5 cannot be considered as part of double-blind design.

^††^Dual phase design; only the RCT part is analyzed in this table.

^†††^Group 1: silkworm weavers given Mori folium extracts (SWE). Group 2: silkworm weavers given placebo (SWP). Group 3: nonsilkworm weavers given Mori folium extracts (NSWE). Group 4: nonsilkworm weavers given placebo (NSWP).

**Table 4:** Korean medical literature on Mori folium.

Author	Published	Name of the book	Relevant ICD-10 category
Unknown	0th–2nd	*Divine Agrarian's Canon of Materia Medica*	R50 fever of other and unknown origin

Ge Hong	3rd	*Eating Your Way to Immortality*	A09 other gastroenteritis and colitis of infectious and unspecified origin T14 injury of unspecified body region

Tao Hongjing	2nd-3rd	*Supplementary Records of Famous Physicians*	T63 toxic effect of contact with venomous animals

Su Jing	7th	*Newly Revised Materia Medica*	R52 pain, not elsewhere classified K59 other functional intestinal disorders

Meng Shen	7-8th	*Materia Medica for Successful Dietary Therapy*	R63 symptoms and signs concerning food and fluid intake

Chen Zangqi	8th	*Chen Zangqi's Materia Medica*	K52 other noninfective gastroenteritis and colitis T14 injury of unspecified body region

Cao Beng	8th	*Four Tones Materia Medica*	K52 other noninfective gastroenteritis and colitis

Rihuazi	10th	*Materia Medica by Rihuazi*	M25 other joint disorders, not elsewhere classified M06 other rheumatoid arthritis R52 pain, not elsewhere classified

Unknown	10–13th	*Illustrated Canon of Materia Medica*	M06 other rheumatoid arthritis

Unknown	14th	*Dan Xi's Words*	R61 hyperhidrosis

Yu Hyo-Tong	15th	*Compilation of Native Korean Prescriptions*	N61 inflammatory disorders of breast G24 dystonia K29 gastritis and duodenitis T30 burn and corrosion, body region unspecified

Liu Wentai	16th	*Collection of the Essential Medical Herbs of Materia Medica*	A09 other gastroenteritis and colitis of infectious and unspecified origin T14 injury of unspecified body region T63 toxic effect of contact with venomous animals

Chén Jiä-Mó	16th	*Coverage of the Materia Medica*	H04 disorders of lacrimal system T63 toxic effect of contact with venomous animals R52 pain, not elsewhere classified M25 other joint disorders, not elsewhere classified K52 other noninfective gastroenteritis and colitis M06 other rheumatoid arthritis T14 injury of unspecified body region

Li Chan	16th	*Introduction to Medicine*	H04 disorders of lacrimal system T63 toxic effect of contact with venomous animals R52 pain, not elsewhere classified M25 other joint disorders, not elsewhere classified K52 other noninfective gastroenteritis and colitis M06 other rheumatoid arthritis T14 injury of unspecified body region

Li Shizhen	16th	*Materia Medica Outline*	R05 cough H54 visual impairment including blindness (binocular or monocular) L67 hair colour and hair shaft abnormalities E14 unspecified diabetes mellitus

Miu Xi-Yong	17th	*Annotations to the Divine Husbandman's Classic of Materia Medica*	R61 hyperhidrosis R52 pain, not elsewhere classified H54 visual impairment including blindness (binocular or monocular) R63 symptoms and signs concerning food and fluid intake L67 hair colour and hair shaft abnormalities K92 other diseases of digestive system

Miu Xi-Yong	17th	*Wide-Rangings Medical Notes from the First-Awakened Studio *	H11 other disorders of conjunctiva

Wu, Youxing	17th	*Ben Cao Sheng Ya Ban Jie*	R50 fever of other and unknown origin

Heo Jun	17th	*Treasured Mirror of Eastern Medicines*	K52 other noninfective gastroenteritis and colitis R52 pain, not elsewhere classified M25 other joint disorders, not elsewhere classified N61 inflammatory disorders of breast L02 cutaneous abscess, furuncle, and carbuncle R61 hyperhidrosis

Lun Zhu	18th	*Poem Collection of Materia Medica *	H04 disorders of lacrimal system L02 cutaneous abscess, furuncle, and carbuncle

Wú Yíluò	18th	*Renewed Materia Medica *	T14 injury of unspecified body region H54 visual impairment including blindness (binocular or monocular) L67 hair color and hair shaft abnormalities H04 disorders of lacrimal system M06 other rheumatoid arthritis E14 unspecified diabetes mellitus R61 hyperhidrosis

Huang Gongxiu	18th	*Truth-Seeking Herbal Foundation*	H54 visual impairment including blindness (binocular or monocular)

Guö Rû-Cóng	19th	*Combined Annotations of Three Experts on the Classic of Materia Medica*	R61 hyperhidrosis

Unknown	19th	*Ben Cao Shu Zheng*	R50 fever of other and unknown origin

Yang Shitai	19th	*An Expoundation on the Origin of the Herbal *	H11 other disorders of conjunctiva

Chen Qirui	19th	*Ben Cao Cuo Yao*	H54 visual impairment including blindness (binocular or monocular) R61 hyperhidrosis H04 disorders of lacrimal system L02 cutaneous abscess, furuncle, and carbuncle

Beijing Institute of Chinese Medicine	20th	*Yao Xing Ge Kuo Si Bai Wei Bao Hua He*	H54 visual impairment including blindness (binocular or monocular) J00 acute nasopharyngitis [common cold] R42 dizziness and giddiness R04 haemorrhage from respiratory passages J02 acute pharyngitis

Szechuan Chinese Materia Medica Editing Committee	20th	*Szechuan Chinese Materia Medica*	H54 visual impairment including blindness (binocular or monocular) J00 acute nasopharyngitis [common cold] H10 conjunctivitis

Shin Gil-Gu	20th	*Shin's Materia Medica*	R50 fever of other and unknown origin R61 hyperhidrosis R52 pain, not elsewhere classified R60 edema, not elsewhere classified T63 toxic effect of contact with venomous animals R63 symptoms and signs concerning food and fluid intake T14 injury of unspecified body region K52 other noninfectious gastroenteritis and colitis R05 cough H54 visual impairment including blindness (binocular or monocular) R61 hyperhidrosis E14 unspecified diabetes mellitus H10 conjunctivitis

Lee Sang-In	20th	*Herbology*	H54 visual impairment including blindness (binocular or monocular) R61 hyperhidrosis E14 unspecified diabetes mellitus R05 cough H10 conjunctivitis T14 injury of unspecified body region R52 pain, not elsewhere classified T63 toxic effect of contact with venomous animals

Nan Jing Traditional Chinese Medical School	20th	*Chinese Medical Great Dictionary*	J00 acute nasopharyngitis [common cold] R51 headache H54 visual impairment including blindness (binocular or monocular) R63 symptoms and signs concerning food and fluid intake R05 cough M06 other rheumatoid arthritis D04 carcinoma in situ of skin L23 allergic contact dermatitis

Lee Sang-In	20th	*Clinical Application of Korean Medical Herbs*	J00 acute nasopharyngitis [common cold] H54 visual impairment including blindness (binocular or monocular)

Zhen Xunying	20th	*Illustrated Chinese Medical Great Dictionary*	J00 acute nasopharyngitis [common cold] R51 headache H10 conjunctivitis H54 visual impairment including blindness (binocular or monocular) R63 symptoms and signs concerning food and fluid intake I10 essential (primary) hypertension R60 edema, not elsewhere classified M06 other rheumatoid arthritis D04 carcinoma in situ of skin L23 allergic contact dermatitis

Nationwide Chinese Medical Herb Compilation Committee	20th	*Nationwide Compilation of Chinese Medical Herbs*	J00 acute nasopharyngitis [common cold] R51 headache H10 conjunctivitis J02 acute pharyngitis

Jiangxi Health and Welfare Ministry	20th	*Jiangxi Herbal Preparation Guideline*	J00 acute nasopharyngitis [common cold] R51 headache R42 dizziness and giddiness H10 conjunctivitis J02 acute pharyngitis

Zhou jinzhong	20th	*Essential in Collection and Use of Chinese Herbs*	J00 acute nasopharyngitis [common cold] R51 headache R42 dizziness and giddiness H10 conjunctivitis J02 acute pharyngitis H04 disorders of lacrimal system

Lin Tongguo	20th	*Practical Chinese Medicine Guidelines for Clinical Syndromes*	J00 acute nasopharyngitis [common cold] R63 symptoms and signs concerning food and fluid intake H54 visual impairment including blindness (binocular or monocular)

Color Illustrated Chinese Medicine Pharmacopoeia Editing Committee	20th	*Color Illustrated Chinese Medicine Pharmacopoeia*	J00 acute nasopharyngitis [common cold] R51 headache R42 dizziness and giddiness H10 conjunctivitis

Editing Committee	20th	*Chinese Herbology*	J00 acute nasopharyngitis [common cold] R42 dizziness and giddiness H10 conjunctivitis

Great Collection of Chinese Medicine Editing Committee	20th	*Great Collection of Chinese Medicine*	J00 acute nasopharyngitis [common cold] R51 headache R42 dizziness and giddiness H10 conjunctivitis

National Korean Medical College Textbook Editing Committee	20th	*Herbology*	J00 acute nasopharyngitis [common cold] R51 headache H10 conjunctivitis

Kim Ho-Chul	21th	*Korean Medicine Pharmacology*	R50 fever of other and unknown origin J00 acute nasopharyngitis [common cold] H10 conjunctivitis H43 disorders of vitreous body

Song and Kim	21th	*Ethnomedicinal Application of Plants in the Western Plain region of North Jeolla Province in Korea*	J02 acute pharyngitis

English translations of author and text names were used whenever available.
